# Acoustic Radiation Force Impulse Elastographic Study of Lung Lesions in Dogs

**DOI:** 10.1111/vru.70031

**Published:** 2025-05-06

**Authors:** Bruna Bressianini Lima, Rafael Kretzer Carneiro, Brenda Santos Pompeu Miranda, Beatriz Gasser, Luiz Paulo Nogueira Aires, Verônica Maria Teixeira de Castro Terrabuio, Ricardo Andrés Ramirez Uscategui, Antônio Carlos Cunha Lacreta Junior, Danuta Pulz Doiche, Gabriela Castro Lopes Evangelista, Marcus Antônio Rossi Feliciano

**Affiliations:** ^1^ Departamento de Clínica e Cirurgia Veterinária Faculdade de Ciências Agrárias e Veterinárias, Universidade Estadual Paulista “Júlio de Mesquita Filho” – FCAV/Unesp Jaboticabal Brazil; ^2^ Departamento de Medicina Veterinária, Centro de Ciências Agroveterinárias Universidade do Estado de Santa Catarina – CAV/UDESC Lages Brazil; ^3^ Instituto de Ciências Agrárias Universidade Federal dos Vales do Jequitinhonha e Mucuri – ICA/UFVJM Unaí Brazil; ^4^ Facultad de Veterinaria y Zootecnia Universidad del Tolima Ibagué Colombia; ^5^ Departamento de Medicina Veterinária, Faculdade de Zootecnia e Medicina Veterinária Universidade Federal de Lavras – FZMV/UFLA Lavras Brazil; ^6^ Departamento de Medicina Veterinária, Faculdade de Zootecnia e Engenharia de Alimentos Universidade de São Paulo – FZEA/USP Pirassununga Brazil

**Keywords:** elastography, shear wave, lung, dog

## Abstract

This study aimed to evaluate the use of acoustic radiation force impulse (ARFI) elastography as a diagnostic tool for lung lesions in dogs. Dogs referred to the Radiology Department of the Veterinary Teaching Hospital between 2020 and 2022 for the detection of lung lesions were included in the study. The characteristics of the lung lesions were assessed using radiography as a screening tool for localization, B‐mode ultrasound for tissue characterization, and subsequently, both qualitative (elastogram grades 1–3) and quantitative (shear wave velocity—SWV) elastographic evaluations. The lesions were classified based on clinical, ultrasound, radiographic, histopathological, and/or cytological findings into the following categories: consolidations, atelectasis, or neoplasms (nodules and masses). Twenty‐six dogs met the eligibility criteria and were included in the study. In some cases, the same dog had more than one type of lesion, resulting in the evaluation of 35 lung lesions: 13 masses, 8 nodules, 8 consolidations, and 4 areas of atelectasis. The quantitative elastographic evaluation revealed lower stiffness in atelectatic lesions (1.48 ± 0.35 m/s) compared with consolidations (2.94 ± 0.64 m/s), nodules (2.85 ± 1.40 m/s), and masses (3.13 ± 1.45 m/s), although no definitive diagnostic cut‐off value was established, due to the limited number of benign lesions. The results suggest that ARFI elastography can be a valuable complementary tool alongside clinical data and conventional imaging techniques in assessing lung lesions in dogs. Future studies with a larger sample size of benign parenchymal lung lesions are needed to further explore the potential of elastography for predicting malignancy.

## 1 Introduction

Dogs with primary or metastatic pulmonary neoplasia may manifest nonspecific clinical signs that can be confused with heart disease, among others. Signs such as chronic cough, exercise intolerance, progressive weight loss, tachypnea, hemoptysis, and cyanosis, often culminate in a late diagnosis of pulmonary neoplasia [1, 2, 3]. Therefore, imaging tests such as radiography and ultrasound are useful for diagnosing, staging, and monitoring these patients [2, 4, 5].

At the radiographic examination, lung metastasis usually presents a structured interstitial pattern commonly related to multiple nodules [6]; this radiographic pattern can be confused with infections (especially fungal), foreign body reactions, hypersensitivity, immune‐mediated diseases, and pulmonary lobe torsion [7]. Depending on the lung condition, various radiographic abnormalities may be present concomitantly, including pleural effusion and other lung pathology patterns, that may reduce the visibility of metastatic pulmonary disease [6].

In these cases, ultrasound can be used to help in the diagnostic process [8, 9], principally when the pleural effusion is present because it makes it possible to distinguish between pulmonary alterations and pleural effusion and visualize peripheral lung lesions, that are inapparent or overlapped by pleural effusion at radiographic examination [5, 9, 10]. Ultrasound can also observe neoplasm behavior during breathing [6, 11] and help to perform fine‐needle aspiration (FNA) and/or percutaneous biopsies [12], with the advantage of performing these biopsies exactly from the site of the lesion and obtaining representative samples [13].

Although lung ultrasound allows an acceptable diagnostic approach to various lung alterations, atelectasis, consolidation, and pulmonary nodules/masses [14], it is still not very accurate for differentiating other abnormalities such as, fungal pneumonia, neoplastic processes, or chronic abscesses [15], often requiring the application of invasive and risky techniques to achieve a definitive diagnosis.

Elastography allows tissue stiffness estimation and could become a noninvasive alternative for differentiating lung lesions in animals. Studies in humans have shown that this technique allows accurate differentiation of necrotic, atelectatic, consolidation, and pulmonary neoplastic lesions [16, 17].

Elastography is developing rapidly. Initially, the technique consisted of applying manual pressure with a transducer to the tissues. The system evaluated the deformation by the pressure generated and presented an image of this deformation (elastogram). By comparing lesion deformation with healthy regions of the evaluated tissue or other tissues, the relative tissue stiffness was determined in percentage. This technique is operator‐dependent, and although the systems have integrated methods for the control and standardization of manual pressure, its reproducibility is not the best [18, 19, 20, 21].

Consequently, the acoustic radiation force impulse (ARFI) elastography technique was developed. The ARFI elastography is based on the emission of an ultrasonic impulse capable of reaching and displacing a target tissue, the system captures the sound waves of this impulse that bounce off the tissue, and estimates the tissue hardness by means of the rebound speed and the wave characteristics, making it much less operator dependent and with better reproducibility. Any elastography techniques used will provide parameters for the subjective, creating a static map (elastogram) of the relative tissue stiffness to be compared with the corresponding B‐mode image; this map is available in color or gray scales. In general, the lighter areas (bluish tones) represent more deformable tissues, in other words, less rigid than the darker areas (reddish tones), and objective (shear wave velocity, pressure force measurement or strain ratio) evaluation of tissue hardness, which is related to the nature of lesions in various tissues, their value (m/s) being directly proportional to their hardness [18, 19, 20, 21].

Based on these precepts, elastography appears to be a promising alternative for the noninvasive diagnosis of lung lesions in dogs, which is a major clinical challenge today and which, to the authors' knowledge, has not been explored to date. For this reason, this study aimed to evaluate ARFI elastography as a tool for the diagnosis of lung lesions in dogs.

## 2 Material and Methods

### 2.1 Ethical Aspects and Cases’ Selection

A prospective cohort study for diagnostic validation was conducted at the “Governador Laudo Natel” Veterinary Hospital of the Faculty of Agricultural and Veterinary Sciences (FCAV), UNESP/Jaboticabal, Brazil, between 2020 and 2022, following approval from the Institutional Ethics Committee (protocol no. 799/21). The owners of the animals enrolled in this study provided written, free, and informed consent, agreeing to the proposed assessments.

Dogs referred to the radiology department for the detection of lung lesions due to clinical signs such as coughing, sneezing, bilateral nasal discharge, dyspnea, abdominal breathing, exercise intolerance, or syncope, as well as those diagnosed with primary malignant neoplasms and referred for the detection of lung metastases, during the aforementioned period, were considered eligible. Inclusion required the presence of visible lung lesions on chest radiography and/or ultrasound, with a definitive diagnosis confirmed either by cytological or histopathological examination of the lesions or pleural fluid or through clinical history that included an oncological diagnosis of primary lesions (e.g., a confirmed diagnosis of primary neoplasm in another organ via histopathology or cytology). There were no restrictions regarding age, gender, breed, size, reproductive status, medication, or previous treatment.

Dogs with lesions located in central or deep lung regions that could not be identified via ultrasound, dogs that were hemodynamically unstable and therefore unable to undergo biopsy, or cases where the owner did not authorize the procedure were excluded from the study.

### 2.2 Thoracic Radiography

Radiographic examinations were performed using a conventional X‐ray machine (Siemens RG150/100 gL), capturing thoracic images in left lateral, right lateral, ventrodorsal, and dorsoventral projections. Images were acquired using AGFA brand computerized imaging plates of various sizes and digitized with an AGFA CR‐30 digital scanner. The digital radiographs were analyzed using the DICOM (Digital Imaging and Communications in Medicine, Rosslyn, VA, USA) software by two veterinarians experienced in diagnostic imaging (BBL), including the chief radiologist with 19 years of expertise in the field (MARF). The radiographic images were archived for subsequent comparison with other variables collected during the study.

### 2.3 Thoracic Ultrasound

The procedure began with a trichotomy of the examination area (chest), and the patients were restrained manually, positioned with their heads facing the monitor and their bodies parallel to the device. To ensure optimal image acquisition, the ultrasound gel, a viscous mixture of water and propylene glycol, was applied to the patient's skin. This gel served as a conductive medium between the skin and the transducer, facilitating smooth gliding and effective ultrasound transmission.

The animals were examined in stationary sternal decubitus, right lateral, and/or left lateral positions, depending on the access required to the ultrasound windows. The dorsal decubitus position was not used to avoid respiratory discomfort in the dogs. Ultrasound images were taken using a SIEMENS ACUSON S2000 device (Siemens, Munich, Germany), equipped with a matrix and a multifrequency linear transducer ranging from 9.0 to 13.0 MHz. A scanning protocol was implemented to evaluate the entire lung by systematically sliding the transducer along the intercostal spaces in the right and left hemithorax. The lungs were assessed in the caudal, perihilar, middle, and cranial regions, as proposed by Assis & Feliciano [15].

The B‐mode ultrasound evaluation focused on identifying alterations, such as the discontinuity or absence of A‐lines, the presence of B‐lines, and consolidated lung areas, which included atelectasis, nodules, masses, or multiple lesions. These alterations were characterized based on their echogenicity (hypoechoic, hyperechoic, or mixed, with the presence of echogenic points, cystic areas, shadowing, or acoustic enhancement), echotexture (homogeneous or heterogeneous), contours (regular or irregular), borders (defined or undefined), number (single or multiple), dimensions (length and width), and the presence or absence of associated pleural effusion. The ultrasound examinations were carried out by a veterinary radiologist with 7 years of experience in veterinary ultrasound (BBL).

### 2.4 ARFI Lung Elastography

Once lung parenchyma lesions were identified during the B‐mode ultrasound examination, the elastography software (Virtual Touch Tissue Imaging Quantification, 2D‐SWE technique) was activated. The lesion was positioned as centrally as possible on the screen, and an elastogram image was captured. When the software captures this image, it displays a colorful quality control bar on the screen; only elastograms with high quality were saved.

Subsequently, the veterinary radiologist performed both qualitative and quantitative evaluations of the stored elastograms. In the qualitative assessment, lesions in the elastogram (color scale map) were classified based on the color as: (1) low stiffness lesions colored in blue and light green; (2) moderately stiff lesions colored in dark green and light yellow; and (3) high stiffness lesions colored in dark yellow and red.

For the quantitative evaluation, five regions of interest (ROIs) measuring 1 × 1 mm were randomly placed on the lesion, ensuring that all areas of the lesion were included. The software then automatically calculated the shear wave velocity (SWV in m/s) for each ROI, providing a quantitative assessment of tissue stiffness.

### 2.5 Cytological and/or Histopathological Analysis

Patients with superficial lung lesions located in the periphery, whose owners authorized the invasive diagnostic approach, underwent FNA using 20 to 27‐gauge needles and/or percutaneous biopsies. For sample collection, the animals were subjected to neuroleptoanalgesia or general anesthesia. After thorough antisepsis of the skin, ultrasound‐guided FNA and/or Tru‐Cut biopsy was performed.

The material collected via FNA was placed on smear slides, fixed, and sent to the Pathology Department for subsequent staining and microscopic evaluation. Biopsy samples obtained using a 16 to 18‐gauge Tru‐Cut needle were fixed in a 10% formaldehyde solution buffered with phosphates (pH 7.4) and sent for routine processing, followed by hematoxylin and eosin staining. The samples were then analyzed under light microscopy by an experienced pathologist, who provided the histopathological report and diagnosis.

In cases where cytological and/or histopathological samples could not be obtained primarily, in patients with primary neoplasms in other organs referred for metastasis detection, or in cases with severe pleural effusion where biopsy was not feasible due to respiratory or circulatory instability, clinical history findings associated with primary tumors, ultrasound and radiographic data, and/or pleural effusion cytochemical analysis were considered. These cases were classified based on the nature of the lesions (as described below), with the assistance and support of the oncology department at the “Governador Laudo Natel” Veterinary Hospital of the School of Agricultural and Veterinary Sciences, UNESP/Jaboticabal, Brazil.

### 2.6 Lesions Classification

The animals with lung lesions included in this study were classified into one of four study groups: masses, nodules, consolidations, or atelectasis, based on the results of histological, cytological, and cytochemical analyses and radiographic/ultrasound characteristics and clinical history.

Lesions suggestive of neoplasia on X‐ray and/or ultrasound (typically ovoid and generally hypoechoic, located immediately below the pleural surface of the lung) were classified based on their size: lesions larger than 3 cm were classified as masses, while those smaller than 3 cm were classified as nodules. Subsequently, within the general lesion description, histopathology, cytology, and clinical analysis results were incorporated to identify the type of neoplasia.

Consolidation and atelectasis were primarily differentiated based on their ultrasonographic appearance [22]. In cases of atelectasis, lung volume is reduced due to collapse caused by increased pleural pressure [23], and its echogenicity varies depending on the amount of air remaining inside the lung: it appears hyperechoic when a significant amount of air is retained in the alveoli and hypoechoic when little air is present [11]. In contrast, lung consolidation does not involve a reduction in lung parenchyma size but instead exhibits echogenicity and echotexture similar to that of the liver (a hepatized lung) [9], due to alveolar spaces being filled with fluid, cellular exudate, or neoplastic cells [5]. The classification process carefully considered the results of the performed examinations and the patient's clinical history.

### 2.7 Statistical Analysis

The statistical analysis was performed using R software (The R Foundation for Statistical Computing, version 4.3.1, USA). Initially, the SWVs obtained from the various regions of interest (ROIs) within each lesion were compared using the Kruskal–Wallis test. If no significant differences were found, the median values of these ROIs were used as the lesion SWV for further analysis.

Subsequently, the lesion SWV and qualitative elastogram grades were compared among the four study groups (masses, nodules, consolidations, and atelectasis) using the Kruskal–Wallis test followed by Dunn's post hoc test. Statistical significance for all tests was set at *p* < .050, and the results are presented as the median ± interquartile range. Other qualitative variables collected will be described in the results section. The statistical analysis was performed by co‐author RARU, a veterinarian, MSc, PhD, with extensive experience (10 years) and training in univariate and multivariate statistical analysis and experimental design.

## 3 Results

### 3.1 Selected Cases

Twenty‐six dogs met the eligibility criteria and were included in the study. In some cases, the same patient had more than one type of lesion studied at the same time, totaling 35 lung lesions assessed, of which 15 were masses, 8 nodules, 8 consolidations, and 4 areas of atelectasis.

Five pleural effusion analyses were carried out, six histopathological examinations, and four cytology, and in the remaining cases, the clinical history associated with imaging findings, such as radiography and ultrasound, was considered, as in the case of metastases, where the main tumors causing metastases included mammary tumors, osteosarcomas, melanomas, and splenic neoplasms.

Of the 35 lesions evaluated, only two can be considered “benign”, one of these in the consolidations group, presenting a clinical history of pneumonia, and another one in the atelectasis group, with effusion analysis conclusive of hemorrhage. The data of the lesions were evaluated, and their characteristics are shown in Table 1

**TABLE 1:** B‐mode ultrasound data, ARFI elastography (qualitative and quantitative), biopsy analysis, and clinical from the patients in this study.

Lung lesions	E.G.	SWV (m/s)	B lines	P.E	Len.	Wid.	Echo.	Ecot.	Borders	Contour	E.S.	C.A	N.	Analysis
**Masses**														
M1	1	3.10	0	0	3.6	4.2	1	2	1	1	1	0	1	Histopathology (carcinoma)
M2	2	4.35	0	1	4.7	4.2	3	2	2	1	1	1	1	Histopathology (adenocarcinoma)
M3	1	1.83	0	1	3.4	2.1	1	1	1	1	0	0	2	P.E (suggestive of lymphoma)
M4	2	3.15	0	0	3.2	4.1	1	1	2	1	0	0	2	Clinical history (metastasis)
M5	3	6.13	0	1	3.4	1.2	3	2	2	1	1	0	1	P.E (suggestive of carcinoma)
M6	1	2.19	1	0	4.98	4.5	1	2	2	1	0	1	2	Histopathology (carcinoma)
M7	1	3.08	0	0	5.3	4.5	3	2	2	1	1	0	1	Cytology (suggestive of carcinoma)
M8	1	2.03	0	0	5.7	3.8	1	2	2	1	0	0	2	Cytology (suggestive of carcinoma)
M9	3	2.82	0	0	5.3	4.6	3	2	2	1	1	1	2	Clinical history (metastasis)
M10	1	3.58	0	1	3.5	4.8	3	2	2	2	0	1	2	Clinical history (metastasis)
M11	1	3.24	0	0	4.5	6.8	3	2	2	2	1	1	2	Histopathology (carcinoma)
M12	3	2.46	0	0	5.4	5.0	1	1	1	1	0	0	2	Clinical history (metastasis)
M13	2	4.41	0	0	8.7	8.2	3	2	2	1	0	1	2	Clinical history (metastasis)
M14	2	1.53	0	1	3.5	2.1	3	2	2	1	1	0	2	Cytology (suggestive of carcinoma)
M15	2	3.49	0	0	5.7	4.1	3	2	2	1	0	0	1	Histopathology (adenocarcinoma)
**Nodules**														
N1	1	3.75	1	0	1.6	1.2	1	1	1	1	0	0	2	Clinical history (metastasis)
N2	2	1.37	0	0	2.3	2	3	2	1	1	0	1	2	Clinical history (metastasis)
N3	1	2.72	0	1	1.5	1.2	3	2	2	2	0	0	2	P.E (epithelial neoplasm cells)
N4	2	1.11	0	0	1.1	1.2	1	2	2	1	0	0	2	Clinical history (metastasis)
N5	2	4.30	0	0	0.4	0.6	1	1	1	1	0	0	2	Clinical history (metastasis)
N6	1	2.97	0	1	1.87	0.8	1	2	1	1	0	0	2	Clinical history (metastasis)
N7	1	3.10	0	0	0.8	0.5	1	1	1	1	0	0	2	Clinical history (metastasis)
N8	1	2.60	0	0	2.2	2.5	3	2	2	1	0	1	2	Clinical history (metastasis)
**Atelectasis**														
A1	1	1.61	0	1			2	1	2	1	0	0	1	P.E (hemorrhagic)
A2	1	1.19	0	1			2	1	2	1	0	0	1	P.E (suggestive of lymphoma)
A3	1	1.55	0	1			2	1	2	1	0	0	1	Histopathology (carcinoma)
A4	1	1.41	0	1			2	1	2	1	0	0	1	Clinical history (metastasis)
**Consolidations**														
C1	1	2.90	0	0			1	2	1	1	1	0	1	Clinical history (pneumonia)
C2	1	2.47	0	0			1	2	1	1	1	0	1	Clinical history (metastasis)
C3	1	3.00	0	0			3	2	2	1	0	1	1	Clinical history (metastasis)
C4	2	3.14	0	0			3	2	2	1	1	0	1	Cytology (suggestive of carcinoma)
C5	1	2.46											1	Clinical history (metastasis)
C6	1	2.98	0	0			1	2	2	1	1	0	1	Clinical history (metastasis)
C7	1	2.46	0	0			1	2	1	1	0	0	1	Clinical history (metastasis)
C8	2	3.26	0	0			1	1	2	1	0	0	1	Clinical history (metastasis)

*Note*: E.G. = Elastogram grade as qualitative elastography, predominant colors: blue and green (1) green and yellow (2) yellow and red (3); SWV—shear wave velocity as quantitative elastography expressed in m/s; Lin. B (B Lines): (0) absent (1) present; P.E (pleural effusion): (0) absent (1) present; Len. = length; Wid. = width; Echo. = echogenicity: (1) hypoechogenic; (2) hyperechogenic; (3) mixed; (4) anechogenic; Ecot = ecotexture: (1) homogeneous; (2) heterogeneous; borders: (1) regular (2) irregular; contour: (1) well defined (2) poorly defined. E.S (echogenic spots): (0) absent (1) present; C.A (cystic areas): (0) absent (1) present; N.: (number of lesions): single (1), multiple (2).

### 3.2 Thoracic Radiography

**FIGURE 1:**
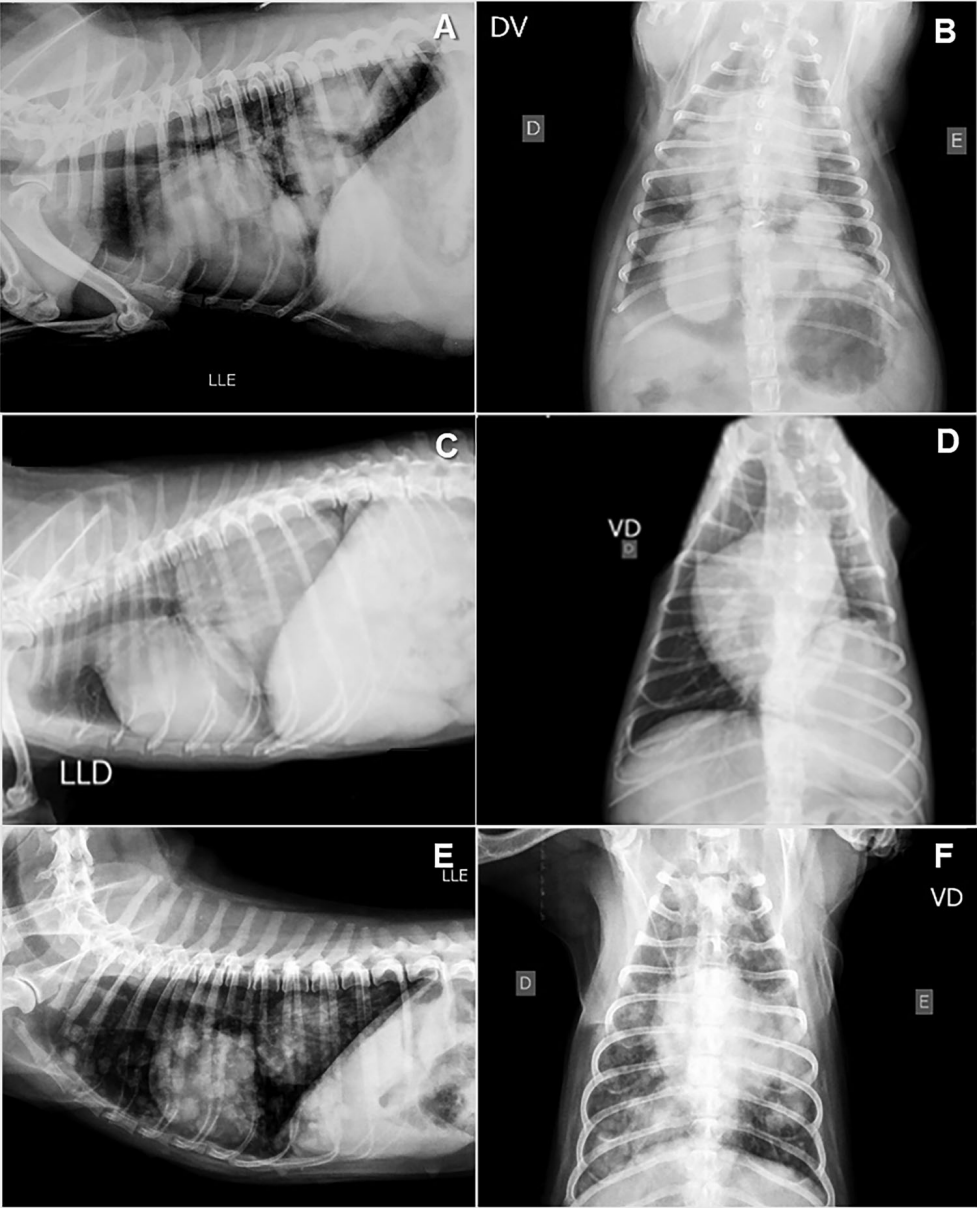
Left lateral (A) and ventrodorsal (B) radiographs of the thoracic region of a dog with a structured interstitial pattern evidenced by multiple lung formations of different sizes, diffusely distributed throughout the parenchyma. Right lateral (C) and ventrodorsal (D) chest radiographs of a dog with a lung formation in the caudoventral (C) and caudomedial (D) regions of the left caudal and accessory pulmonary lobes, causing deviation of the left main bronchus and its respective branches, visualized in ventrodorsal projection. Left lateral (E) and ventrodorsal (F) chest X‐rays reveal the presence of numerous diffusely distributed pulmonary nodules. There is also a larger formation in the left caudal lobe.

Masses were the predominant lesions observed on radiographic examination (n = 15), showing structured interstitial lung patterns. The solitary masses (*n* = 5) had a predilection for the caudal lobes (Figure 1C,D); while the others (*n* = 10) had multiple formations in different lung lobes (Figure 1A,B,E,F). The nodules larger than 0.7 cm had sufficient radiopacity for identification on radiographic examination and did not predominate in any specific lung lobe, being visible in different lung portions. Radiographic samples showing examples of the lesions assessed in this study and their respective descriptions are shown in Figure 1.

Regarding consolidations, the lesions showed an increase in lung radiopacity and little definition and often could not be distinguished from masses, nor could the possibility of consolidation and pulmonary masses at the same time be ruled out. In these cases, ultrasound was essential for better detail. Atelectasis was characterized exclusively by ultrasound due to the presence of pleural effusion.

### 3.3 B‐Mode Ultrasound

**FIGURE 2:**
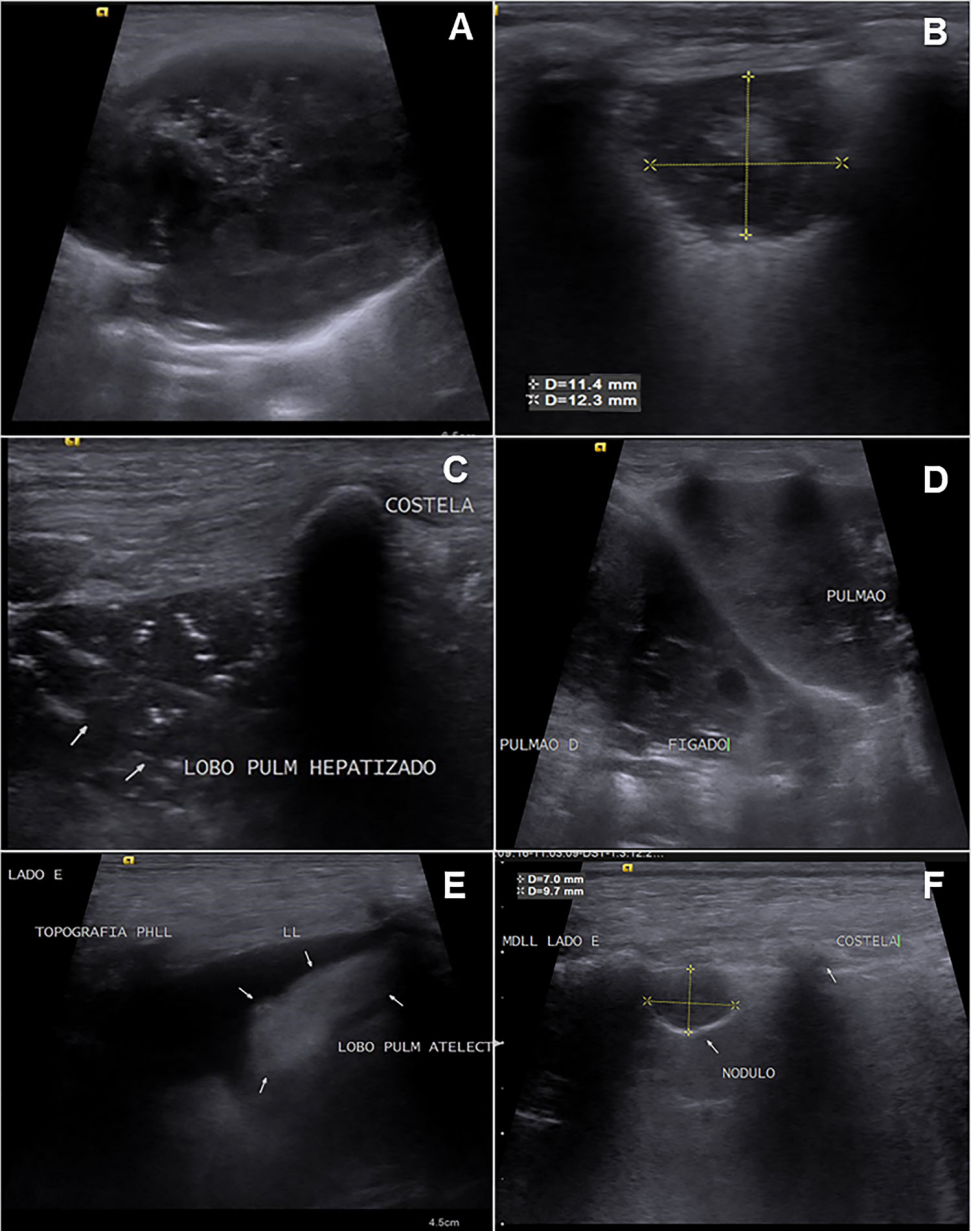
A, Lung mass, heterogeneous in appearance, with echogenic spots and small cavitary areas in the center of the lesion, with mixed echogenicity, irregular margins, and well‐defined contours. B, Nodular formation in the lung, subpleural, predominantly hypoechogenic, with slightly irregular margins and well‐defined contours, slightly heterogeneous, measuring approximately 11.4 × 12.3 mm. C, lung consolidation, with a hepatized appearance, showing hyperechogenic punctate echoes, corresponding to air bronchograms, giving the lesion a heterogeneous appearance and hypoechogenic lung parenchyma. D, Lung also shows pulmonary consolidation, and the liver parenchyma can be seen to the left of the image, for comparison purposes, with a very similar appearance to the pulmonary lobe, hence the term hepatization, when the lung is well consolidated. E, pulmonary atelectasis, showing the left lung lobe in peri hilar topography, with a triangular shape, hyperechogenic, homogeneous, supernatant pleural effusion present, (F) nodule in the left lung parenchyma in the medial region, subpleural, hypoechogenic, homogeneous, with regular margins and well‐defined contours, measuring approximately 7 × 9.7 mm.

The consolidation group showed predominantly hypoechogenic lungs, with echogenic spots in the consolidated lobe, characterizing the air bronchograms and giving a heterogeneous appearance (Figure 2).

Atelectasis was characterized when pleural effusion was present and appeared as triangular, hyperechogenic, homogeneous areas with slightly irregular margins and well‐defined contours, overlying the pleural effusion (Figure 2).

Most of the nodules were hypoechogenic with regular margins and well‐defined contours. Most of the masses showed mixed echogenicity (62.5%), the other masses were hypoechogenic, and when the echotexture was studied, 81.3% had a heterogeneous appearance. However, the heterogeneous appearance was also a common feature in the groups of consolidations and nodules (Figure 2).

### 3.4 ARFI Lung Elastography

**FIGURE 3:**
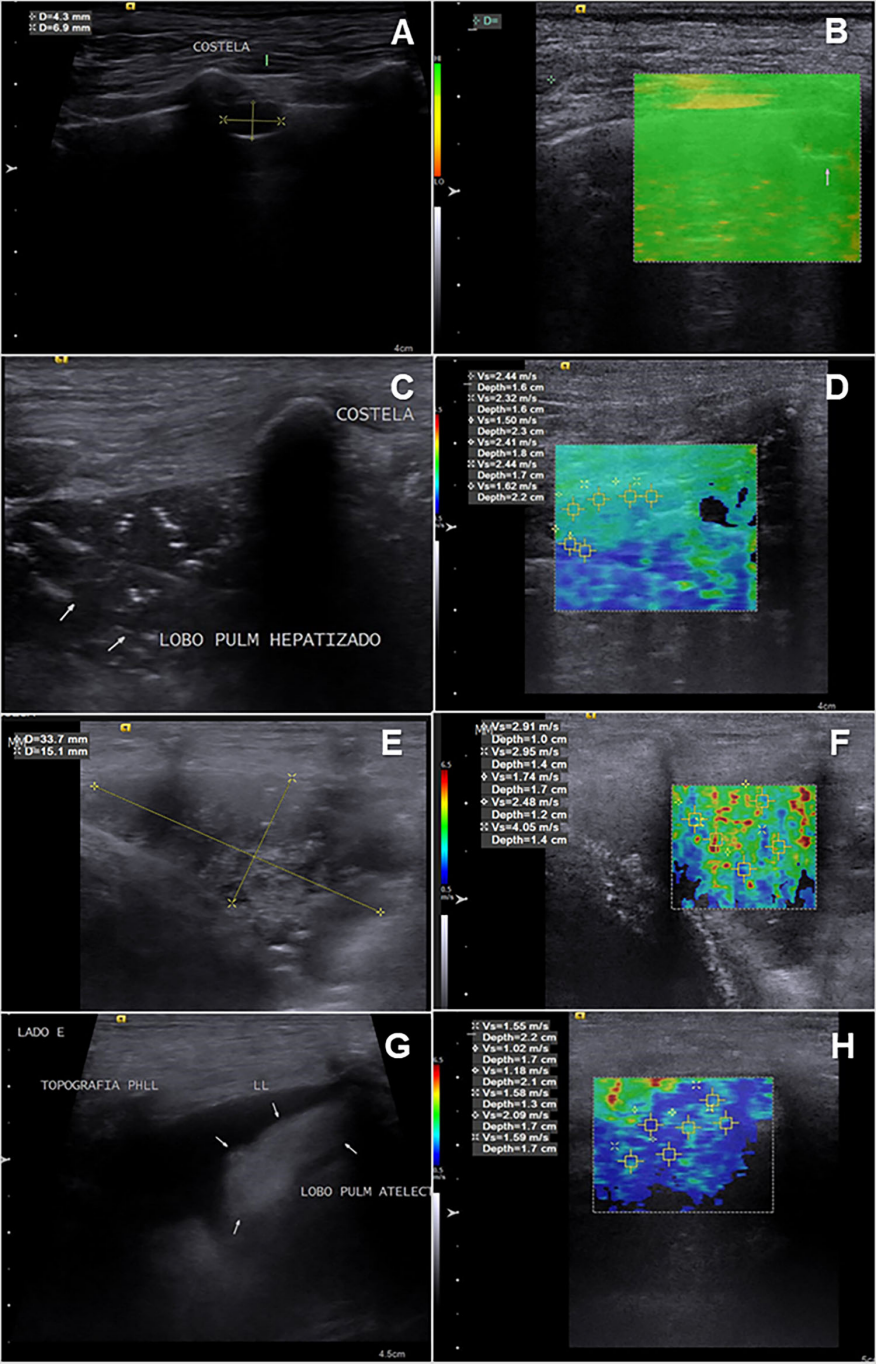
A, C, E, G, Corresponding to b‐mode ultrasound. B, D, F, H, Same lesion, but with ARFI mode activated, performing qualitative (color map) and quantitative elastographic analysis with ROIs (region of interest) positioned on the lesions to obtain numerical SWV values in m/s. Images were obtained using SIEMENS ACUSON S2000 equipment. A, Presence of a nodular formation in the lung, measuring approximately 4.3 × 6.9 mm. B, Same formation with ARFI mode activated in its quality map, where green areas correspond to an ideal site for evaluation and orange areas to poor quality for the elastographic study. C, Pulmonary consolidation, with a hepatized appearance. D, Same consolidation as image C, with ARFI mode activated, it is possible to see the elastogram in shades of blue and green representing soft tissues. After the qualitative assessment, a quantitative assessment was obtained (SWV m/s). E, Presence of a formation in the lung, measuring approximately 33.7 × 15.1 mm. F, Formation corresponding to image (E), with ARFI mode activated. G, Corresponds to pulmonary atelectasis, showing a triangular‐shaped, hyperechogenic, homogeneous pulmonary lobe in perihilar topography, supernatant to pleural effusion. H, Corresponds to the elastogram of image G, in its qualitative and quantitative study.

**FIGURE 4:**
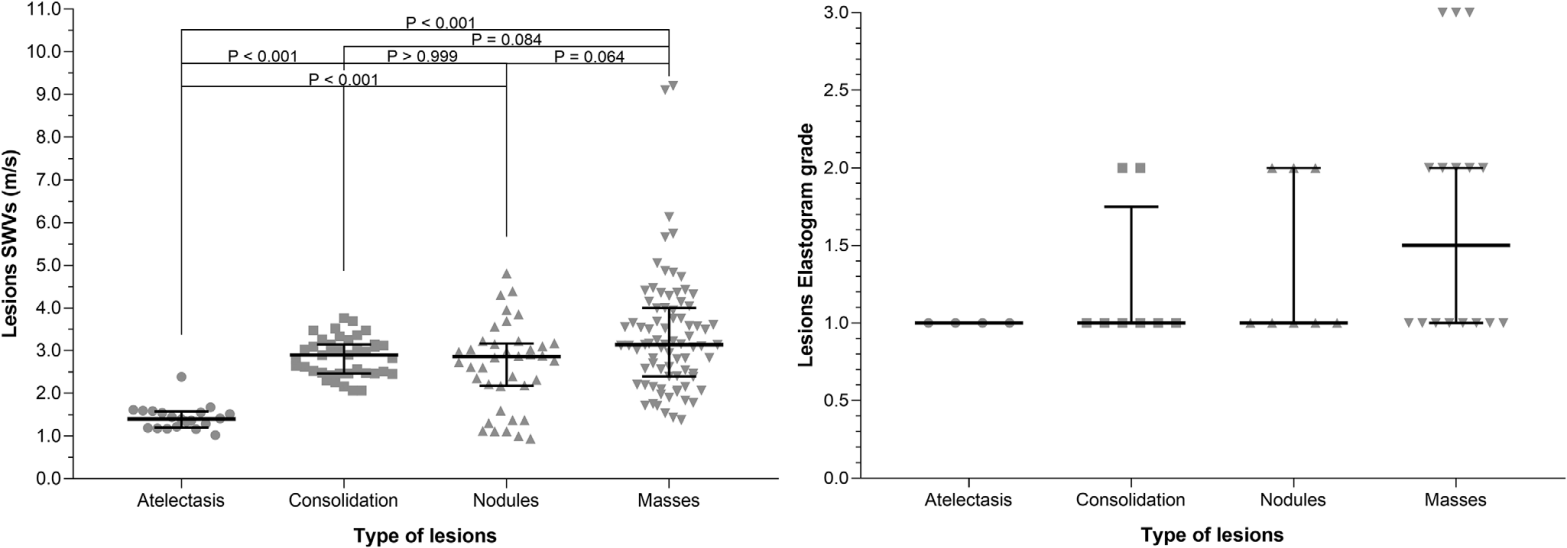
Graphical representation of the elastographic evaluation, quantitative (shear wave velocity—SWV—m/s; left) and qualitative (elastogram grades 1–3; right). The grey points represent each of the measurements performed on the lung lesions (including all ROIs evaluated in the quantitative evaluation), the thicker horizontal line is the median, and the bars are the interquartile range. Additionally, in the quantitative evaluation (left), the *p*‐values of the Dunns post hoc comparisons were included.

Examples of B‐mode images of lung lesions and their corresponding elastographic studies are shown in Figure 3. The results of the quantitative and qualitative elastography analysis are represented in Figure 4. The SWVs obtained from the different ROIs selected in each lesion studied were similar (*p* = .3831). Comparing the types of lesions, the SWV of the atelectatic lesions (1.48 ± 0.35 m/s) was lower (*p* = .0187) than the SWV of consolidation (2.94 ± 0.64 m/s), nodules (2.85 ± 1.40 m/s), and masses (3.13±1.45 m/s). In qualitative elastography, the elastogram grades resulted similar between the types of lesions (*p* = .5541), atelectatic lesions (1.00 ± 0.00), consolidation (1.00 ± 0.75), nodules (1.00 ± 1.00), and masses (1.50 ± 1.00).

## 4 Discussion

This study primarily evaluated the applicability of qualitative and quantitative ARFI elastography analysis in differentiating atelectatic, consolidated, and neoplastic (nodules and masses) lung lesions. The key finding demonstrated a gradual increase in tissue stiffness, as assessed quantitatively, from atelectasis to consolidations, nodules, and masses, with statistical significance observed only when comparing atelectasis with other lesion types. Additionally, ultrasound characteristics evaluated in B‐mode, along with the radiographic features of each lesion type, were also analyzed.

Radiographs played a crucial role in this study, preceding ultrasound evaluations to determine the location and type of lesions and to establish the appropriate starting point for ultrasound examination, as described by Mattoon & Nyland [5]. However, in cases where pleural fluid was present alongside lung disease, radiographic examination was unable to characterize the type of lung lesion. In such instances, ultrasound provided greater detail of the intrathoracic structures, with the fluid serving as an acoustic window that enabled excellent visualization of most structures, which aligns with findings by Hassan et al. [24]. Additionally, ultrasound facilitated real‐time guidance for pleural interventions [25]. All lung surface masses detected on radiographs were easily identified and characterized using ultrasound. However, due to the small number of benign lesions (*n* = 2), the ultrasonographic, elastographic, and radiographic characteristics could not be assessed for their ability to predict malignancy.

Qualitative elastography of the studied lesions did not demonstrate diagnostic capability, as there was a predominant “soft” appearance on the elastogram, indicated by the frequent presence of blue and green colors, representing less rigid tissue across all evaluated groups. This characteristic was expected in the nodule, consolidation, and atelectasis groups, as these lesions generally exhibit lower stiffness compared with masses, according to Lim et al. [16]. However, the predominance of softer areas in masses may be attributed to the presence of necrotic regions. As noted by Daleck & De Nardi [20], lung neoplasms can exhibit a mixed tissue composition, with softer areas (blue and green) associated with necrotic processes, and stiffer regions (yellow and red) corresponding to viable parenchymal portions of the tumor on the elastogram.

Regarding quantitative elastography, atelectasis was the softest (1.48 ± 0.35 m/s) lesion compared with the others, aligning with the findings of Lim et al. [16]. Although their study utilized compressive elastography rather than ARFI, they reported significant differences in strain ratio values among the lesion types: (2.51 ± 1.14) for atelectasis, (19.98 ± 15.59) for consolidation, and (36.19 ± 20.18) for tumoral lesions. The authors employed CT as the gold standard for lesion characterization.

The relative softness of atelectasis is believed to be due to its distinct tissue characteristics. Alveoli are surrounded and separated by an extremely thin layer of connective tissue [26], which contains blood capillaries that lack connective tissue in their walls. In addition to capillaries, the alveolar interstitium contains elastic and collagen fibers produced by interstitial fibroblasts [27]. These morphological characteristics of the atelectatic process, combined with the elastographic findings of this study, support the high deformability observed in atelectasis (alveolar collapse). This aligns with the observations of Konofagou [28], who stated that the presence of collagen fibers, elastin, water, and fat molecules contributes to the specific elasticity and rigidity attributes of this tissue when subjected to external forces.

The median SWV of nodules (2.85 ± 1.40 m/s), masses (3.13 ± 1.45 m/s) (malignant neoplasms), and consolidations (2.94 ± 0.64 m/s) in this study resulted in statistically similar, which contrasts with the findings of Lim et al. [16], where neoplasms exhibited greater stiffness compared with consolidations. Our hypothesis for this result is that, of the eight consolidations analyzed in this study, only one was benign, attributed to pneumonia, while the remaining cases resulted from neoplastic infiltrates. A similar observation was reported by Cole et al. [29], who found that pulmonary neoplasia was the most common cause of consolidation in a population of dogs with respiratory disorders. It is believed that the tissue composition associated with the infiltrate contributed to this finding, as pulmonary consolidation involves the filling of alveoli with various materials, including exudate, transudate, blood, neoplastic cells, and other components [30].

In many cases, the small nodules were still partially aerated, suggesting that the tissue was not fully structured. This could be another reason for their similar rigidity compared with lung consolidations. According to Konofagou [28], the mechanical properties of tissues are closely related to their molecular composition and structural organization, which in turn influence the tissue's stiffness. This aligns with our observations of the small nodules.

In this study, the group of tumors was separated according to the presentation of nodules and masses. In medicine, the size and density of nodules are criteria that directly affect the prognosis and diagnosis of patients, and nodular structures are classified according to their density [31]. In our study, formations smaller than 7 mm did not have a visible tissue composition on the radiograph, and it was not possible to identify them, according to Thrall [6]. However, formations smaller than 7 mm, when located on the periphery of the lung or when there was pleural effusion, were well characterized on ultrasound [15].

Although it is not possible to make a B‐mode assessment of the density of nodular structures, ultrasound is an important screening tool for assessing small nodules, smaller than 7 mm, whose identification is limited on radiography [32] and when associated with the elastography technique, it is possible to assess tissue characteristics, providing information on tissue morphology through the variation in elasticity in each type of tissue [33, 34], thus providing a field for future studies that evaluate cut‐off values for predicting malignancy in pulmonary nodules according to tissue stiffness in elastography, just as it is already possible to predict malignancy according to nodule density in CT.

The masses had greater numeric tissue rigidity than the other lesions studied (3.13 ± 1.45 m/s), and this is directly related to their morphological characteristics. According to Daleck & De Nardi [20], every neoplasm has a stroma and parenchyma, and the stroma is made up of fibrous and vascular connective tissue, providing support and nutrition for the parenchyma. This fibrous connective tissue is probably the cause of the increased tissue stiffness observed on quantitative elastography. However, this pattern was not predominant in the masses evaluated, as there was also a wide variation in stiffness along the length of the formations studied, and “soft” areas were also identified, that is, areas of low tissue stiffness. This phenomenon can be explained by the fact that in fast‐growing malignant neoplasms, the vascular stroma may not be proportional to the volume of the parenchyma, so angiogenesis occurs at a slower rate than the proliferation of the parenchyma and, therefore, large areas of the neoplasm suffer ischemic (coagulation) necrosis or infarction [20], thus creating areas of less rigidity in certain tumor regions.

This study had some limitations that should be noted: (1) The inability to establish cut‐off values for predicting malignancy due to the small sample size of benign lesions, as malignant lung neoplasms were much more prevalent in this study. (2) The challenge of obtaining histopathological diagnoses in all cases would have been the gold standard but limited in some cases due to nonauthorization by the owners or hemodynamic instability of the patient. (3) The presence of fluid collections in large masses with cavitary areas proved to be a limitation. In some portions of these masses where small fluid collections were present, the elastography quality map indicated poor quality, signaling an unsuitable area for sample collection. Therefore, only the solid, parenchymal portions, which had good quality, were studied. (4) Normally, the tissue of a healthy organ serves as a comparison when performing elastographic studies of possible pathologies; however, in the case of the lung, an aerated organ healthy tissue reflects ultrasound waves, making it unsuitable for comparison with pathological tissues. (5) Additionally, respiratory movements did not pose a limiting factor for elastographic evaluation in our study.

In this study, the thoracic ultrasound protocol was essential for assessing lesions and making quick decisions, such as draining the pleural effusion. Elastography after B‐mode ultrasound is a noninvasive exam that does not require anesthesia, costs less than computerized tomography and MRI, does not contain ionizing radiation, and there are already portable devices with elastography software, making it a more accessible exam modality that can be taken to the patient, avoiding unnecessary travel in critically ill patients. We hope that in the near future, through more research in the area, elastography will become a tool with high diagnostic accuracy in the chest, helping critically ill patients; the aim of this manuscript was not to study elastography to replace other imaging tests but rather to present lung elastography as a complementary method to existing tests.

## 5 Conclusion

The quantitative elastographic evaluation revealed lower stiffness in atelectatic lesions (1.48 ± 0.35 m/s) compared with consolidations (2.94 ± 0.64 m/s), nodules (2.85 ± 1.40 m/s), and masses (3.13 ± 1.45 m/s), although no definitive diagnostic cut‐off value was established, due to the limited number of benign lesions. The results suggest that ARFI elastography can be a valuable complementary tool alongside clinical data and conventional imaging techniques in assessing lung lesions in dogs. Future studies with a larger sample size of benign parenchymal lung lesions are needed to further explore the potential of elastography for predicting malignancy.

## List of Author Contributions

### Category 1

(a) Conception and design: Bruna Bressianini Lima, Rafael Kretzer Carneiro, Brenda Santos Pompeu Miranda, Beatriz Gasser, Luiz Paulo Nogueira Aires, Marcus Antônio Rossi Feliciano
(b) Acquisition of data: Bruna Bressianini Lima, Marcus Antônio Rossi Feliciano
(c) Analysis and interpretation of data: Bruna Bressianini Lima, Rafael Kretzer Carneiro, Brenda Santos Pompeu Miranda, Beatriz Gasser, Luiz Paulo Nogueira Aires, Verônica Maria Teixeira de Castro Terrabuio; Ricardo Andrés Ramirez Uscategui, Antônio Carlos Cunha Lacreta Junior, Danuta Pulz Doiche, Gabriela Castro Lopes Evangelista; Marcus Antônio Rossi Feliciano

### Category 2

(a) Drafting the article: Bruna Bressianini Lima, Rafael Kretzer Carneiro, Luiz Paulo Nogueira Aires, Gabriela Castro Lopes Evangelista; Marcus Antônio Rossi Feliciano
(b) Revising article for intellectual content: Bruna Bressianini Lima, Rafael Kretzer Carneiro, Brenda Santos Pompeu Miranda, Beatriz Gasser, Luiz Paulo Nogueira Aires, Verônica Maria Teixeira de Castro Terrabuio; Ricardo Andrés Ramirez Uscategui, Antônio Carlos Cunha Lacreta Junior, Danuta Pulz Doiche, Gabriela Castro Lopes Evangelista; Marcus Antônio Rossi Feliciano

### Category 3

(a) Final approval of the completed article: Bruna Bressianini Lima, Rafael Kretzer Carneiro, Brenda Santos Pompeu Miranda, Beatriz Gasser, Luiz Paulo Nogueira Aires, Verônica Maria Teixeira de Castro Terrabuio; Ricardo Andrés Ramirez Uscategui, Antônio Carlos Cunha Lacreta Junior, Danuta Pulz Doiche, Gabriela Castro Lopes Evangelista; Marcus Antônio Rossi Feliciano

### Category 4

(a) Agreement to be accountable for all aspects of the work in ensuring that questions related to the accuracy or integrity of any part of the work are appropriately investigated and resolved: Bruna Bressianini Lima, Rafael Kretzer Carneiro, Brenda Santos Pompeu Miranda, Beatriz Gasser, Luiz Paulo Nogueira Aires, Verônica Maria Teixeira de Castro Terrabuio; Ricardo Andrés Ramirez Uscategui, Antônio Carlos Cunha Lacreta Junior, Danuta Pulz Doiche, Gabriela Castro Lopes Evangelista; Marcus Antônio Rossi Feliciano

## Conflicts of Interest

The authors declare no conflicts of interest.

## Previous Presentation or Publication Disclosure

This manuscript has not received prior publication and is not under consideration for publication elsewhere.

## Reporting Checklist Disclosure

No reporting checklist was used.

## Data Availability

Data supporting the results are available from the corresponding author upon reasonable request.
